# Normal range of motion at the hip show different pressure behavior in the lateral and acetabular compartments: a cadaveric investigation

**DOI:** 10.1186/s40634-022-00450-6

**Published:** 2022-02-05

**Authors:** Marc-Olivier St-Pierre, Félix-Antoine Lavoie, Marion Hoffmann, Mickaël Begon, Antony Bertrand-Grenier, Stéphane Sobczak

**Affiliations:** 1 Chaire de recherche en anatomie fonctionnelle, Université du Québec à Trois-Rivières, 3351, boul. des Forges C.P. 500, Trois-Rivières, QC G8Z 4M3 Canada; 2 Département d’anatomie, Université du Québec à Trois-Rivières, 3351, boul. des Forges C.P. 500, Trois-Rivières, QC G8Z 4M3 Canada; 3 Département des Sciences de l’activité physique, Université du Québec à Trois-Rivières, 3351, boul. des Forges C.P. 500, Trois-Rivières, QC G8Z 4M3 Canada; 4 École de Kinésiologie et des Sciences de l’Activité Physique, Faculté de Médecine, Université de Montréal, Campus Laval, 1700 rue Jacques Tétreault, Laval, QC H7N 0B6 Canada; 5 Institut de Génie Biomédical, Faculté de Médecine, Université de Montréal, Campus Laval, 1700 rue Jacques Tétreault, Laval, QC H7N 0B6 Canada; 6 Département de chimie, biochimie et physique, Université du Québec à Trois-Rivières, 3351, boul. des Forges C.P. 500, Trois-Rivières, QC G8Z 4M3 Canada; 7 CIUSSS de la Mauricie-et-du-Centre-du-Québec, Centre hospitalier affilié universitaire régional, 1991 Boulevard du Carmel, Trois-Rivières, QC G8Z 3R9 Canada

**Keywords:** Hip, Pressure, Clinical assessment, Acetabular cavity, Capsular chamber, Cadaveric specimen

## Abstract

**Purpose:**

The techniques used previously to assess intracapsular pressures did not allow the assessment of pressure variations in both compartments throughout the entire range of motion without puncturing the capsular tissue. Our hypothesis was that the intra-capsular pressure would be different in the lateral and acetabular compartment depending on the movement assessed.

**Methods:**

Eight hip joints from four cadaveric specimens (78.5 ± 7.9 years) were assessed using intra-osseous tunnels reaching the lateral and acetabular compartments. Using injector adaptors, 2.7 ml of liquid were inserted in both compartments to simulate synovial liquid. Optic pressure transducers were used to measure pressure variations. We manually performed hip adduction, abduction, extension, flexion and internal rotation at 90° of flexion.

**Results:**

Hip extension and internal rotation show the highest intra-capsular pressures in the lateral compartment with increases of 20.56 ± 19.29 and 19.27 ± 18.96 mmHg, respectively. Hip abduction and hip internal rotation showed depressurisations of − 16.86 ± 18.01 and − 31.88 ± 30.71 mmHg in the acetabular compartment, respectively. The pressures measured in the lateral compartment and in the acetabular compartment were significantly (*P* < 0.05) different for the hip abduction, 90° of flexion and internal rotation. Pressure variations showed that maximum intracapsular fluid pressures in the lateral compartment occur at maximum range of motion for all movements.

**Conclusion:**

As an increase in pressure may produce hip pain, clinician should assess pain at maximum range of motion in the lateral compartment. The pressure measured in the acetabular compartment vary depending on the hip position. The movements assessed are used in clinical practice to evaluate hip integrity and might bring pain. The pressure variations throughout the entire range of motion are a relevant information during hip clinical assessment and might help clinicians to better understand the manifestations of pain.

## Introduction

An increase in intracapsular fluid pressures (ICFP) in the hip joint is strongly associated with hip pain [7, 14]. The intracapsular pressure is, on one hand, affected by the volume of fluid [23] and, on the other hand, by the position of the joint [18, 21, 23]. Although previous studies reported the ICFP in-vivo and in vitro, different methodological aspects might have affected the results.

Previous studies used an anterior approach and a piezoelectric transducer, perforating the iliofemoral ligament and reaching the mid-portion of the femoral neck, to measure intracapsular pressure in the lateral compartment [7, 14, 18, 21, 23]. This technique did not take into consideration the anatomical characteristics of the joint capsule as being a hermetic volume having pressurization capacities. The acetabular labrum is an important anatomic structure of the hip joint and allows some liquid exchanges from the acetabular to the lateral compartments [4]. However, previous studies reported intracapsular pressure measurements only in the lateral compartment [7, 14, 18, 21, 23]. The depressurization in the acetabular compartment seems to improve the stabilization of the hip during distraction [17]. To date, no study has reported the presence of suction effect within the acetabular compartment during classic hip movements. Levels of depressurization might highlight its importance in classic motion and not only during hip mobilization. Therefore, intracapsular pressures should be assessed simultaneously in the lateral and acetabular compartments to respect the anatomical characteristics and limit the damage to the capsular tissue [3].

ICFPs have been reported at given angles or at the maximal range of motion without providing its association with hip kinematics. ICFPs were assessed in different positions such as hip flexion, extension, internal rotation and external rotation in neutral position, abduction and adduction [7, 14, 21, 23]. Hip flexion had been shown to decrease the intracapsular pressure in the lateral compartment when compared to the neutral position with up to an 81% decrease [23]. Other hip movements such as hip extension, internal rotation in neutral position, abduction and adduction brought up to a 5-fold increase when compared to hip flexion.

The capsular tissue and more precisely the synovium, within the lateral compartment and around the femoral head ligament continuing on the fat of the acetabular fossa, contains nociceptive fibers [11]. Previous studies stated the link between pain and an increase pressure in the lateral compartment [7, 14]. The synovium might also be irritated due to pressure fluctuations. However, to date, no studies report pressure fluctuations in this compartment during hip clinical assessment. Therefore, the assessment of pressure in the acetabular compartment is important due to the possible head ligament and fat pad’s synovium irritation during normal range of motions.

The aim of this study is to provide an assessment ICFP simultaneously in the lateral and acetabular compartments without perforating the capsular tissue during the following movements: 90° of flexion, extension, abduction, adduction and internal rotation at 90° of flexion. More specifically, we reported (1) the ICFP at the maximum range of motion and (2) the ICFP of both compartments throughout the movement. Our general hypothesis was that the intra-capsular pressure would be different in the lateral and acetabular compartments depending on the movement assessed. More precisely, the first hypothesis was that hip extension would bring the highest pressure in the lateral compartment by bringing capsular tension. Therefore, a previous study has stated that hip extension bring the highest pressure in the lateral compartment [23]. The second hypothesis was that hip internal rotation would bring the largest changes in the acetabular compartment due to the head motions within the acetabular cavity during mutli-planar motions.

## Methods

### Population

**Fig. 1:**
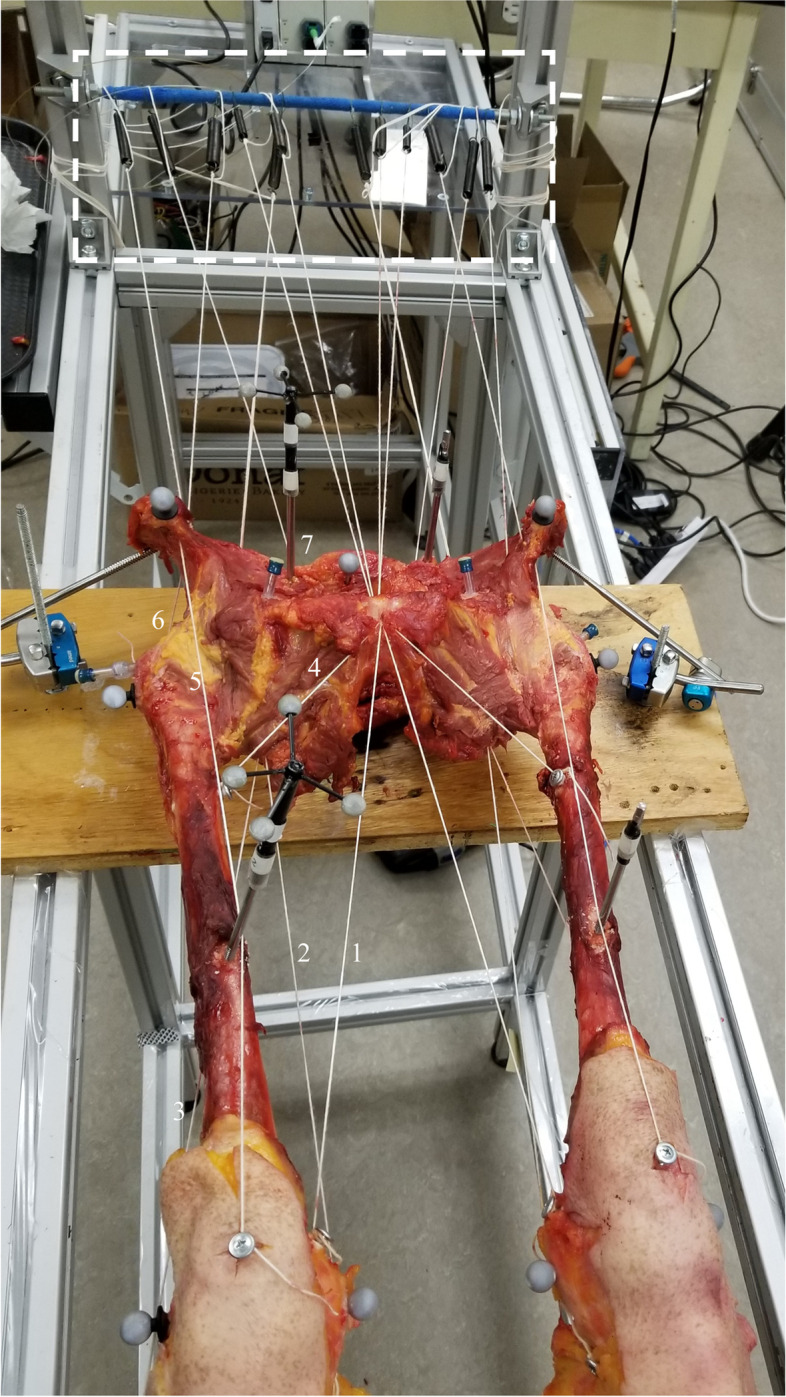
Proximal view of the lower limb

Left and right lower limbs from four fresh-frozen cadaveric specimens (two males and two females aged of 78.5 ± 7.9 years) were used (*n* = 8). The exclusion criteria were any surgical procedure regarding the lower back or limb, unproper range of motion and severe osteoarthritis. Also, specimens were excluded if any range of motion at the hip was particularly limited. The pelvis was taken off cadavers at the L4-L5 junction. While the pelvis was kept intact to improve the stability of lower limbs on the experimental frame, the specimen was skeletonized from the pelvis to the distal femur while preserving the capsular ligaments (Fig. 1). Legs (tibia) were retained to replicate the in-vivo clinical assessment. Each lower limb was moved across a normal range of motion to ensure that subjects had no clinically detectable abnormalities and/or any impaired range of motion. Since hip osteoarthritis affects intracapsular pressure [18], osteoarthritis level [19] was evaluated by a chiropractor using anteroposterior radiographs. OA has been evaluated using an antero-posterior view of hip joints. The parameters were focal distance: 100 cm and 80 kV [2] using a Mobile Capacitor X-ray Generator (model: SMR-16, SEDECAL, Rio de Janeiro). A chiropractor, with a radiological license, assessed this evaluation. Following this assessment, selected hips had low to moderate levels of osteoarthritis (Table 1).

**Table 1:** Radiographic characteristics obtained from anterior-posterior radiological assessment

Specimens	Radiographic characteristics				
	OA	DJS	Sub. Cyst.	Art. Surf. Ch.	Osteo. Pres. & Loc.
1	1	Inferior	No	No	Yes^c^
2	1	No	No	Yes^a^	No
3	1	No	No	No	Yes^d^
4	1	No	No	No	Yes^d^
5	2	Inferior and medial	No	Yes^b^	Yes^d^
6	2	Medial	No	Yes^1^	Yes^d^
7	0	No	No	No	No
8	0	No	No	No	No

*OA* Tönnis Grade (0 = no osteoarthritis, 1 = low, 2 = moderate, 3 = high), *DJS* Decrease Joint space, *Sub. Cyst.* Subchondral cyst, *Art. Surf. Ch*. Articular surface changes, *Osteo. Pres. & Loc*. Osteophyte presence and localization.

^a^Slight degenerative change of the acetabulum

^b^CAM type change

^c^Slight change of the acetabular margin

^d^Upper acetabular margins

Bolts, inextensible ropes, and traction springs were used to simulate in-vivo muscle passive tension during hip intra-articular pressure assessment. Note that this mechanism did not intent to evaluate the muscle moment arms. The following muscle tensions were simulated: gluteus medius and minimus, rectus femoris, hamstrings, adductor magnus, pectineus and piriformis. Proximal and distal tendons of each lower limb muscle were dissected. Lines of action were recreated using a bolt at the distal insertion of the muscle and a hole at the proximal attachment to pass inextensible wires and reached the traction springs (Fig. 1). The relative strength of each spring was proportional to the muscle cross-sectional area [9]. The inextensible wires were attached to traction springs when the lower limbs were placed in an anatomical position [16]. In this manner, the tension increases if the traction springs lengthen and decrease if the distal attachment is getting closer to the proximal attachment.

Pelvis was firmly fixed to the testing table. The pelvis was set in an anatomical position and fixed using two screws passing through the second and third sacral vertebrae and reaching the wooden plate underneath. To ensure a solid fixation, two external fixates were drilled through each iliac bone (diameter: 5 mm, length: 20 mm). The femurs were held parallels to the floor without any external or internal rotations using a mold holding the ankles.

Inextensible ropes and springs are shown in the white dotted rectangle. Muscles are presented as follows:

- (1) Adductor Magnus, (2) Semi-tendinous, (3) Biceps Femoris, (4) Pectineus, (5) Rectus femoris, (6) Gluteus Medius, (7) Piriformis (placed posteriorly).

### Intracapsular pressure assessment

**Fig. 2:**
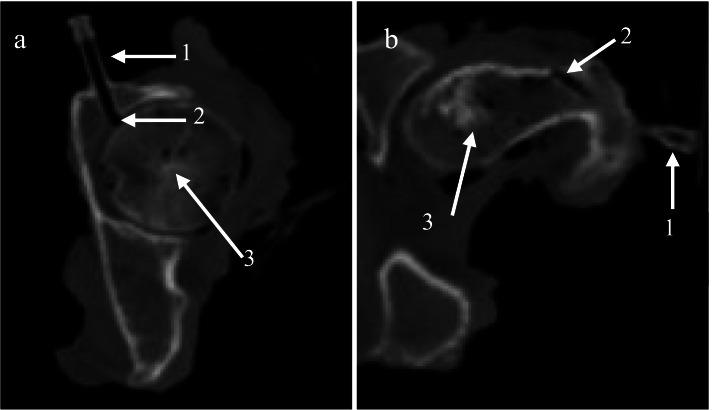
**a**, **b** Axial (superior) view using computed tomography of the intra-osseous tunnels. Small arrows show the injector chamber (1), intra-osseous tunnel entrance (2) and the femoral head (3)

**Fig. 3:**
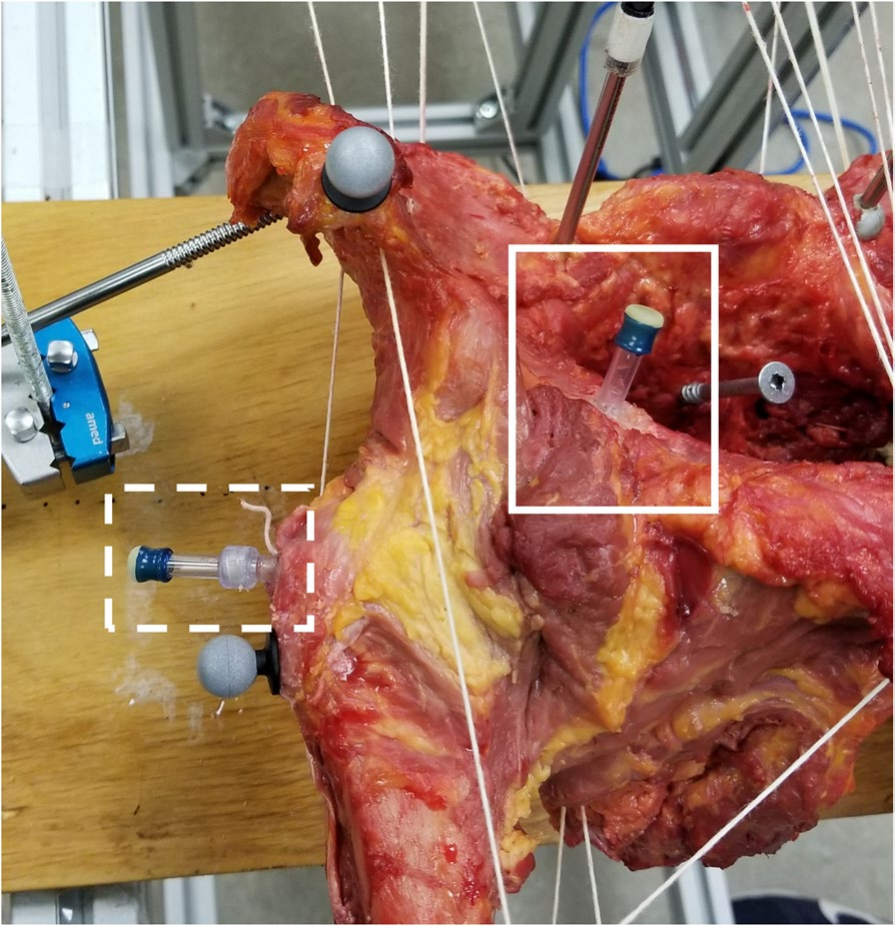
The intraosseous tunnel entrances with their injector chamber*.* Medial and lateral tunnels are shown in the rectangle with solid and dotted lines, respectively

Two intraosseous tunnels that reach the two hip joint compartments were created to limit possible alterations of the pressurization capacities of the capsular tissue. The acetabular tunnel passed through the coxal bone with an anteroposterior direction to reach the upper portion of the acetabular compartment (Fig. 2a). The entrance of the acetabular tunnel was medial to the acetabular borders. The acetabular tunnel was confirmed by performing a lateral distraction of the femoral head and hearing the hip suction. The lateral tunnel passed through the greater trochanter with a lateromedial angulation to emerge medially to the intertrochanteric line (Fig. 2b). One injector chamber was placed at the entrance of each tunnel (Fig. 3). A small wooden rod was inserted in the lateral tunnel. Thereafter, the anterior part of the capsule was palpated to feel the wooden rod, confirming the entrance in the hip capsule. Following the CT-scan imaging, it was possible to confirm both tunnel entrances. The distal part of the injector chamber was threaded and screwed into the bone to ensure a strong hermetic junction. Tissue glue (3 M vetbond™ Tissue Adhesive, St-Paul, MN, USA) was used to seal the injector chamber to the bone. The injector adapters were filled with a mixture of 2.7 ml canola oil and latex to ensure a good signal transmission from the pressure transducers and avoid leakage by small arteries. This amount was used to simulate the amount of synovial liquid in the hip joint in-vivo [13].

Pressure variations were collected during the entire range of motion using two optic pressure transducers (FPI-HR-2, range ± 300 mmHg, accuracy ±1 mmHg, Fiso Technologies, Quebec, Canada) placed into both injector chambers. The pressure transducers were inserted in the injector adapter using 18-gauge needles. Movements such as hip flexion and extension were performed to make sure that each pressure transducer provided pressure variations. Before the beginning of the testing, the pressure transducers were zeroed in neutral position.

### Kinematic assessment

Six cameras (Prime^X22^, Optitrack, NaturalPoint Inc., Corvallis, OR, USA) were set around the testing area. The testing table was placed in the center of the field after the calibration. Three-dimensional kinematic was evaluated using clusters of four passive markers placed bilaterally on each of the following bones: ilium, femur and tibia (Fig. 1). Lower limbs with clusters of markers were scanned by computed tomography (Siemens, SOMATOM definition, Munich, Germany). Medical imaging protocols were standards: pitch = 1.5D, slice intervals: 1 mm, slice thickness: 1 mm. These settings allow the connection between bones and clusters. Based on computed tomography images, lower limbs were segmented in Amira® Software (Amira 5.3, Berlin, Germany). The meshes were linked with a motion tracking data file obtained from the Optitrack system. Pressure variations for both compartments were synchronized with the lower limb kinematics and analyzed together in Matlab (MathWorks, Version: R2020b, Natik, Massachusetts, USA). The orientation of the hip joint in the 3D Euclidean space was calculated using Euler angles with a z-x-y Cardan sequence as recommended by the ISB [22], which represent flexion (+), abduction (+) and external rotation (+), respectively. The measurement error of the Optitrack cameras is 0.05°.

The following movements were performed three times in a randomized order on each lower limb: 90° of flexion, extension, adduction, abduction and internal rotation at 90° of hip flexion. Pressures were measured at the end range of motion for hip extension, abduction, adduction and internal rotation. For hip flexion, 90 degrees represents the end range of motion. The same assessor performs all movements to simulate the clinical assessment from a trained clinician.

### Statistical analysis

The reliability of the pressure variations was evaluated on one hip with at test-retest design with one-hour interval. The same assessor performed every movement. Reliability was evaluated using Pearson’s correlation (multiple coefficients) for both compartments in within (three repetitions) and between sessions and presented using means and standard deviations. The Pearson correlation results were interpreted as follows: 0.00–0.10 as negligible correlation, 0.10–0.39 as weak correlation, 0.40–0.69 as moderate correlation, 0.70–0.89 as strong correlation and lastly, 0.90–1.00 as very strong correlation [15]. We also report the range of motion variations, means and coefficient of variations, across sessions 1 and 2 and the between sessions variation. These variations were measured on one hip with a 1 h interval.

Descriptive statistics for the dependent variable (pressure) such as means and standard deviations were reported for each movement and compartment. The dependant variable did not reach data normality following Shapiro-Wilk test. Therefore, intra-capsular pressures were compared using univariate Kruskal-Wallis tests followed by Bonferroni test to sort differences between movements. Eta-squared was reported regarding significant difference following the Kruskal-Wallis test. Wilcoxon test was used to compare each compartment within each movement. Cohen size effect has also been reported for each Wilcoxon test comparison. Power has been added to each statistically significant difference. The overall significant level was set at 0.05.

To provide an average pressure-range profile, the data of each specimen was expressed (%) with respect to their maximal range of motion and pressure and interpolated using third-order- polynomials. This procedure was performed in order to limit the difference in total range of motion between each specimen. With this procedure, it is possible to compare each specimen with their own maximal range of motion by an limiting the effect between low and high range of motion. The statistical analyses were performed using SPSS (IBM SPSS Statistics 25.0).

## Results

The within session correlation showed a coefficient over 0.90 except for the acetabular compartment in extension showing a correlation of 0.80 ± 0.16 (Table 2). The between session correlation showed a coefficient over 0.84 except for the hip flexion showing a coefficient of 0.62 (Table 2).

**Table 2:** Pearson’s correlation values for the within and between session assessment (Mean ± SD)

	Compartments	ADD	ABD	EXT	F90	IR
Within Session	Lateral	0.98 ± 0.02	0.91 ± 0.07	0.99 ± 0.01	0.98 ± 0.01	0.99 ± 0.01
	Acetabular	0.98 ± 0.01	0.90 ± 0.09	0.80 ± 0.16	0.99 ± 0.01	0.96 ± 0.03
Between Session	Lateral	0.96	0.99	0.96	0.94	0.99
	Acetabular	0.98	0.84	0.94	0.62	0.98

*ADD* Adduction, *ABD* Abduction, *EXT* Extension, *F90* Flexion 90°, *IR* Internal rotation

For all ranges of motion, standard deviations were below 3 degrees (Table 3). The within session CV were below 6% except for internal rotation. The between session variations were lower than 5.5% except for adduction (10.4%) and internal rotation (15.9%) (Table 3).

**Table 3:** Mean, standard deviation and coefficient of variation for within and between session range of motion during adduction, abduction, extension, 90° of flexion, internal rotation

Movements	Session 1		Session 2		Between session	
	Mean (°) ± SD	CV (%)	Mean (°) ± SD	CV (%)	Mean (°) ± SD	CV (%)
**ADD**	18.5 ± 1.0	5.2	15.5 ± 0.8	1.8	17.0 ± 1.8	10.4
**ABD**	23.9 ± 1.4	5.8	24.4 ± 1.1	4.7	24.1 ± 1.3	5.2
**EXT**	15.9 ± 0.9	5.5	15.5 ± 0.9	5.6	15.7 ± 0.9	5.5
**F90**	90.4 ± 1.2	1.3	90.9 ± 2.9	3.2	90.7 ± 2.4	2.5
**IR**	18.6 ± 0.7	3.9	14.7 ± 2.4	16.1	16.6 ± 2.7	15.9

*ADD* Adduction, *ABD* Abduction, *EXT* Extension, *F90* Flexion 90°, *IR* Internal rotation, *SD* Standard deviation, *CV* Coefficient of variation

In the lateral compartment, hip extension showed the highest intracapsular pressure (20.56 ± 19.29 mmHg) while hip adduction showed the lowest pressure in this compartment (4.38 ± 4.28 mmHg). No significant differences in pressure at maximal range of motion between movements for the lateral compartment.

In the acetabular compartment, hip extension showed the highest pressure with 16.31 ± 13.71 mmHg and internal rotation showed the largest depressurization with − 31.88 ± 30.71 mmHg (Table 4). A Kruskal-Wallis test showed that there was a statistically significant difference in pressure between movement in the acetabular compartment (H = 24.150, *P* < 0.001, η^2^ = 0.06). The pressure measured in abduction were significantly lower than in extension (*P* < 0.01) and adduction (*P <* 0.01). Thereafter, the pressure measured in internal rotation were significantly lower than adduction (*P* < 0.01) and extension (*P* < 0.01).

**Table 4:** Means and standard deviations for the intracapsular pressure at maximum range of motion

Compartments	ADD	ABD	EXT	F90	IR	*P* value
Lateral	4.38 ± 4.28	12.31 ± 13.89	20.56 ± 19.29	11.25 ± 6.68	19.27 ± 18.96	*0.093*
Acetabular	9.63 ± 9.29	−16.86 ± 18.01	16.31 ± 13.71	−2.49 ± 11.21	−31.88 ± 30.71	***< 0.001***
*Wilcoxon P value*	*0.21*	0.02*	*0.67*	*0.138*	0.01*	
*Effect size (d)*	*−0.70*	*1.33*	*0.26*	*1.21*	*1.41*	
*Power*	*–*	*0.95*	*–*	*–*	*0.97*	

Values are in mmHg

*ADD* Adduction, *ABD* Abduction, *EXT* Extension, *F90* Flexion 90°, *IR* Internal rotation. Significant level was set at 0.05. (*) Significant difference *p* < 0.05

Two out of five movements (ADB and IR) showed a significant difference between pressures measured in the lateral and acetabular compartments. These differences in abduction and internal rotation showed a strong effect size with respectively 1.33 and 1.41. The power was respectively 0.95 and 0.97 from these differences. Hip extension, flexion and adduction presented no significant difference between both compartments.

**Fig. 4:**
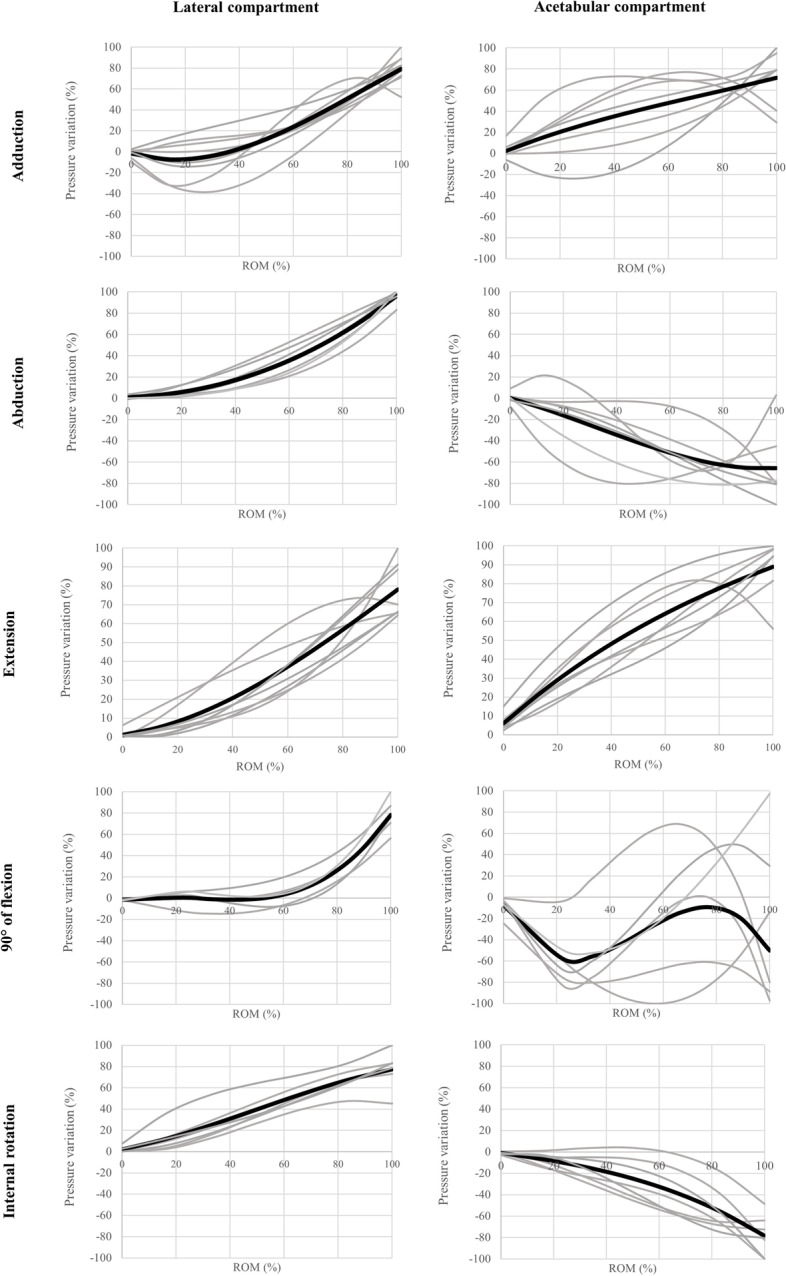
Pressure variations. X axis: range of motion (%), Y axis: intracapsular pressure variations (%)*.* Black curves represent the average curve and curves for all specimens are in grey

All pressures and range of motion are reported in the percentage to normalize variation across every specimen (Fig. 4). The lateral compartment presented an increase in pressure while the hip reached its maximum range of motion. Hip flexion presented little to no increase in the lateral compartment below 60% of the total range of motion. In the acetabular compartment, the pressure increased in hip adduction and extension while the hip reached its maximal range of motion. Hip abduction and internal rotation showed a depressurization in the acetabular compartment. Concerning hip flexion, the pressure decreased in the first portion of the movement (< 30°) and increases passing this point. However, this movement presents high variability as shown by the reliability assessment.

## Discussion

The main findings are that hip extension increases pressure in both compartments of the hip, internal rotation creates an important depressurization in the acetabular compartment while increasing it in the lateral compartment. Therefore, hip flexion, more precisely at 45°, showed a low increase in pressure. The lateral compartment pressure increase is associated with an increase in range of motion for all movements tested. In the acetabular compartment, the pressure increased for both, hip adduction and extension. A depressurization was observed in the acetabular compartment for hip abduction and internal rotation. These results confirm our hypothesis that hip extension brings the highest pressure in the lateral compartment and internal rotation shows the greatest changes in the acetabular compartment. Although high intra-capsular pressure is not a linearly link with pain, pain is important information in clinical assessment and every possible source of pain should be highlighted and understood. The nociceptive fibers in the capsular ligaments and intra-articular ligament are usually unresponsive in normal range of motion [11]. However, during an inflammatory state, such as transient synovitis [20] or idiopathic arthritis [6], they might be sensitive in normal range of motion causing them to discharge and signaling pain at the joint [11]. This sensitivity marks the importance of assessing pressure in both compartments in normal range of motion during hip clinical assessment. Therefore, this is the first study to assess pressure during the entire range of motion without puncturing the capsular tissue. Hip joints were tested as practiced during clinical assessment and this presents a great transfer to clinical settings.

This is the first study that describes hip joint pressure variations for both compartments during a simulated hip clinical assessment. We measured simultaneously the ICFP in both compartments without damaging the capsular tissue, contrary to previous studies [14, 18, 21, 23]. Unlike these studies, muscle tensions were recreated to simulate the in-vivo passive tension to address a problem previously stated [23]. In fact, these authors reported that the resection of all muscles crossing the hip joint might modify the joint behavior [23]. Beforehand, our study assessed the within and between assessment reliability of the pressure variations that were good to excellent in all movements except for hip flexion in the acetabular compartment. Loosening of the capsular ligaments could partly explain this lower correlation [8].

The pressure in the lateral compartment has been assessed previously [14, 18, 23]. In our study, hip extension showed the highest pressure in the lateral compartment with a mean pressure of 20.56 ± 19.29 mmHg. Previous studies have measured, before arthroplasty, pressures of 26 mmHg and 15.80 ± 33.00 mmHg during hip extension [14, 18]. The discrepancies between the absolute values could be explained by different methodological characteristics. First, previous studies used a piezoelectric transducer in an in-vivo experimental model, damaging the capsular tissue. Despite no study has assessed the impact of puncturing the capsular tissue, the hip joint is a hermetic volume and should be assessed as such. Second, it is known that the amount of synovial liquid found in the lateral compartment affects intracapsular pressures [23]. As a result, Robertsson et al. have reported a mean liquid aspiration of 6 ml. This higher volume might partly explain their higher pressure (26 mmHg) compared to our study (20.56 ± 19.29 mmHg).

Hip abduction shows a pressure of 12.31 ± 13.89 mmHg in the lateral compartment in our study. A previous study reported a pressure of 4.80 ± 6.50 mmHg [23]. Both studies have been performed using an in-vitro experimental model. The only major methodological difference comes from the addition of passive muscle tension in our study. However, we can’t state if this difference totally explains the disparity in pressure measurements. Further studies could assess the impact of muscle passive tension on intra-capsular pressures.

Hip internal rotation in 90° of flexion is usually used in clinical settings to assess underlying problems such as femoroacetabular impingement or hip osteoarthritis [1, 10]. This range of motion can cause pain in the hip. Although the possibility of an increase in pressure can be the source of pain, no study, to this date, has assessed the pressure in the lateral compartment during this movement. Hip internal rotation in 90° of flexion brings a mean pressure of 19.27 ± 18.96 mmHg. The comparison can be made with internal rotation in hip extension [23]. In this study, the intra-capsular pressure in the lateral compartment were 10.10 ± 4.80 and 30.50 ± 42.80 mmHg with 2 and 4 ml of liquid injected, respectively. The main difference comes from the capsular tension in extension and flexion. During hip flexion, capsular tissue is released, and this characteristic decreases the intra-capsular pressure [12]. Also, the amount of liquid in our study (2.7 ml) was lower than the pressure measured with 4 ml of liquid (30.50 ± 42.80 mmHg).

The pressure in the lateral compartment seems to be correlated with the higher tension found in the capsular tissue [12]. Clinicians should pay particular attention to pain at the end of the movement as pain is linked with higher intracapsular pressure in this compartment [7, 14]. However, clinicians should keep in mind that pain can come from different sources such as bony modifications, labral tears or, synovial liquid quality.

The acetabular and lateral compartments are only separated by the labrum, although no study has studied pressure during hip clinical assessment in the acetabular cavity. The labrum controls the exchanges of synovial liquid from the acetabular compartment to the lateral aspect of the hip [5]. Also, the acetabular labrum helps to maintain a negative pressure within the acetabular cavity to stabilize the femoral head [17]. A previous study has assessed the pressure in both compartments during simulated walking motion in cadaveric specimens [4]. Our study brings new insight on the pressure during clinical assessment in both compartments simultaneously.

Regarding the pressure variations, the intracapsular pressures in the acetabular compartment show greater variability compared to the lateral compartment. By its smaller volume, the acetabular compartment is more sensitive to small fluctuations of liquid or femoral head movements. The pressure in the acetabular compartment in flexion shows the highest variability when compared to other movements. The hip capsular ligament loosens in flexion (> 30°) bringing micro-motions of the femoral head in the acetabular compartment bringing more uncontrolled pressure variations [8].

Hip abduction and internal rotation showed an important depressurization at the maximum range of motion. The femoral head lateralization at the end of these two movements might explain this depressurization [15]. Also, the internal rotation depressurization could be explained by the cantilever effect between the femoral neck and the anterior part of the acetabular wall. This contact might create a lever effect acting on the femoral head, creating a decoaptation of the femoral head. In comparison, hip adduction and extension increase the pressure in the acetabular compartment with the latero-medial displacement of the femoral head.

This is the first study showing the possibility to assess the pressure in both compartments without damaging the capsular tissue during simulated clinical assessment. Intracapsular pressures, in both compartments, are affected by hip positions. The increases of pressure in the lateral compartment are correlated with a larger range of motion for all movements. In the acetabular compartment, the pressure increases for hip adduction and extension. A depressurization was observed for hip abduction and internal rotation. For hip flexion, the pressure in the acetabular compartment showed large variations in comparison to the lateral compartment. Thereafter, the continuous kinematic assessment permit to observe pressure variations throughout the movement, an important aspect for clinicians.

The use of cadaveric specimens makes it difficult to transfer these new results directly into clinical settings. However, these results could improve the understanding of pain at the hip joint. As stated previously, pain and pressure are not linearly related. Synovium irritation caused by an increase pressure in the lateral compartment has been assessed previously [7, 14]. Our result showed that this impact on the synovium might also be present in the acetabular compartment due to pressure variations in the latter. Each source of pain at the hip should be clearly understood to better assess the hip joint.

The main limitation of this study is the absence of the physiological muscle passive tension (in-vivo) which may differ from our mechanism based on springs and inextensible ropes. The low number of specimens might also present a limitation especially for the hip flexion showing greater variability. However, we found large size effects for the statistical differences between compartments within each test. The specimens used in this study were elderly (> 70 years old). Although the radiographic assessment was used to exclude specimens with high levels of hip osteoarthritis, we did not assess the possible presence of labral tears. In fact, labral tears might decrease the efficiency of the labrum to control liquid exchange from the acetabular compartment. This study was based on the link between increase intra-capsular pressure in the hip joint and hip pain. However, hip pain is not solely caused by an increase in pressure. Despite this limitation, these new results might help clinicians to look out to end range of motion and pain at the hip joint.

## Conclusion

The pressures reported in this study state that it is possible to assess intra-capsular pressure without altering the capsular tissue and assessed the hip as a whole in cadaveric specimens. Intracapsular pressures, in both compartments, are affected by the hip position. The lateral compartment pressure increases are associated with an increase in range of motion for all movements tested. In the acetabular compartment, the pressure increased for both, hip adduction and extension. A depressurization was observed in the acetabular compartment for hip abduction and internal rotation. The pressure in the acetabular compartment showed large variations in comparison to the lateral compartment for hip flexion. This study might help clinicians to have a better knowledge of pressure distribution, refining the hip clinical assessment. As observed in our study, certain movements bring higher pressure variations and these movements might be highlighted in clinical settings. Further studies are needed to understand the importance of pressure changes in the genesis of hip pain for different hip pathologies.

## Data Availability

The datasets generated and/or analysed during the current study are not publicly available due to complication of analysis and large data tables but are available from the corresponding author on reasonable request.
